# A systematic review of feasibility studies promoting the use of mobile technologies in clinical research

**DOI:** 10.1038/s41746-019-0125-x

**Published:** 2019-06-06

**Authors:** Jessie P. Bakker, Jennifer C. Goldsack, Michael Clarke, Andrea Coravos, Cynthia Geoghegan, Alan Godfrey, Matthew G. Heasley, Daniel R. Karlin, Christine Manta, Barry Peterson, Ernesto Ramirez, Nirav Sheth, Antonia Bruno, Emilia Bullis, Kirsten Wareham, Noah Zimmerman, Annemarie Forrest, William A. Wood

**Affiliations:** 1 Philips, Monroeville, PA USA; 2 Clinical Trials Transformation Initiative, Durham, NC USA; 3 Digital Medicine Society (DiMe), Boston, MA USA; 4 monARC Bionetworks, San Francisco, CA USA; 5 Queen’s University Belfast, Belfast, UK; 6 Elektra Labs, Boston, MA USA; 7 Harvard-MIT Center for Regulatory Science, Boston, MA USA; 8 Patient and Partners, Madison, CT USA; 9 Northumbria University, Newcastle upon Tyne, UK; 10 GlaxoSmithKline, Stevenage, UK; 11 HealthMode, New York, NY USA; 12 Independent Consultant, Philidelphia, PA USA; 13 Independent Consultant, Charlotte, NC USA; 14 Evidation Health, San Mateo, CA USA; 15 MicroMedicine, Inc., Waltham, MA USA; 16 Ross University School of Medicine, Bridgetown, Barbados; 17 23andMe Inc., Mountain View, CA USA; 18 PRA Health Sciences, Blue Bell, PA USA; 19 Icahn School of Medicine at Mount Sinai, New York, NY USA; 20 University of North Carolina, Chapel Hill, NC USA

**Keywords:** Clinical trials, Medical research

## Abstract

Mobile technologies, such as smart phone applications, wearables, ingestibles, and implantables, are increasingly used in clinical research to capture study endpoints. On behalf of the Clinical Trials Transformation Initiative, we aimed to conduct a systematic scoping review and compile a database summarizing pilot studies addressing mobile technology sensor performance, algorithm development, software performance, and/or operational feasibility, in order to provide a resource for guiding decisions about which technology is most suitable for a particular trial. Our systematic search identified 275 publications meeting inclusion criteria. From these papers, we extracted data including the medical condition, concept of interest captured by the mobile technology, outcomes captured by the digital measurement, and details regarding the sensors, algorithms, and study sample. Sixty-seven percent of the technologies identified were wearable sensors, with the remainder including tablets, smartphones, implanted sensors, and cameras. We noted substantial variability in terms of reporting completeness and terminology used. The data have been compiled into an online database maintained by the Clinical Trials Transformation Initiative that can be filtered and searched electronically, enabling a user to find information most relevant to their work. Our long-term goal is to maintain and update the online database, in order to promote standardization of methods and reporting, encourage collaboration, and avoid redundant studies, thereby contributing to the design and implementation of efficient, high-quality trials.

## Introduction

An increasing number of clinical trials are being designed in which mobile technology—including smart phone applications, wearables, ingestibles, implantables, and other mobile platforms containing sensors—are being used to capture data of interest to trial stakeholders.^1–4^ Rapidly evolving technology within the last several years has allowed for more powerful algorithms (software) to convert the data that are detected by the sensors (hardware) into clinically meaningful endpoints (outcomes).^5^ For example, technology worn at the wrist might include an accelerometer, and various algorithms may then be applied to the acceleration signal to generate estimates of total sleep time, steps per day, and other endpoints. In addition to digitizing existing endpoints, mobile technologies can be used to develop novel endpoints.

Potential advantages of trials that adopt mobile endpoints include: real-time data capture and analytics; less frequent study visits; the ability to capture day-to-day variability by collecting data continuously; the availability of objective endpoints to complement patient- and clinician-reported outcomes; increased measurement precision and therefore smaller samples; and the ability to collect data that are more likely to reflect habitual, real-world experiences of trial participants.^6–8^

Although mobile endpoint collection may have several potential advantages, guidance is needed, such as the new framework issued by the U.S. Food and Drug Administration (FDA) to promote development of digital tools (https://www.fda.gov/NewsEvents/Newsroom/FDAInBrief/ucm626166.htm) for investigators to make decisions about which technology is most suitable for a particular trial, and what methodology ensures trials are conducted as efficiently as possible.^9,10^ The Clinical Trials Transformation Initiative (CTTI), a public-private partnership co-founded by Duke University and the FDA, has recently issued four sets of recommendations and resources intended as a comprehensive guide to improving clinical trial quality and efficiency through appropriate use of mobile technology. Topics covered include the development of novel endpoints; design and implementation of decentralized trials; the application of mobile technology in clinical trials; and the optimization of mobile clinical trials by engaging patients and sites (https://www.ctti-clinicaltrials.org/programs/mobile-clinical-trials). One strong CTTI Mobile Technology recommendation is for investigators to conduct small feasibility/pilot studies before launching a clinical trial, with the overall aim of reducing risk by assessing sensor accuracy, developing and/or validating algorithms, optimizing data quality, identifying unanticipated challenges, exposing weaknesses of the selected system, enhancing participant experience, and satisfying user engagement. To catalog the breadth of feasibility studies and facilitate the use of these data to enable the development of clinical trials that will use mobile technologies, we conducted a systematic scoping review of the literature, with the overall goal of compiling a living database of feasibility studies. In doing so, our objectives were to promote standardization of feasibility methods; encourage collaboration among investigators and sponsors working in this area; demonstrate the value of feasibility data publication; and avoid the development of redundant studies. The online database derived from the data identified in this review is intended to support the efficient and effective adoption of mobile technologies in clinical research by creating a single, searchable, up-to-date resource that gives users easy access to existing knowledge. The objectives of this paper are therefore to describe the methodology of our systematic scoping review, summarize key trends that emerged from the identified studies, and discuss future directions for maintenance of an online database of feasibility studies designed to advance the science and ultimately the adoption of mobile endpoints.

## Results

### Screening

**Fig. 1:**
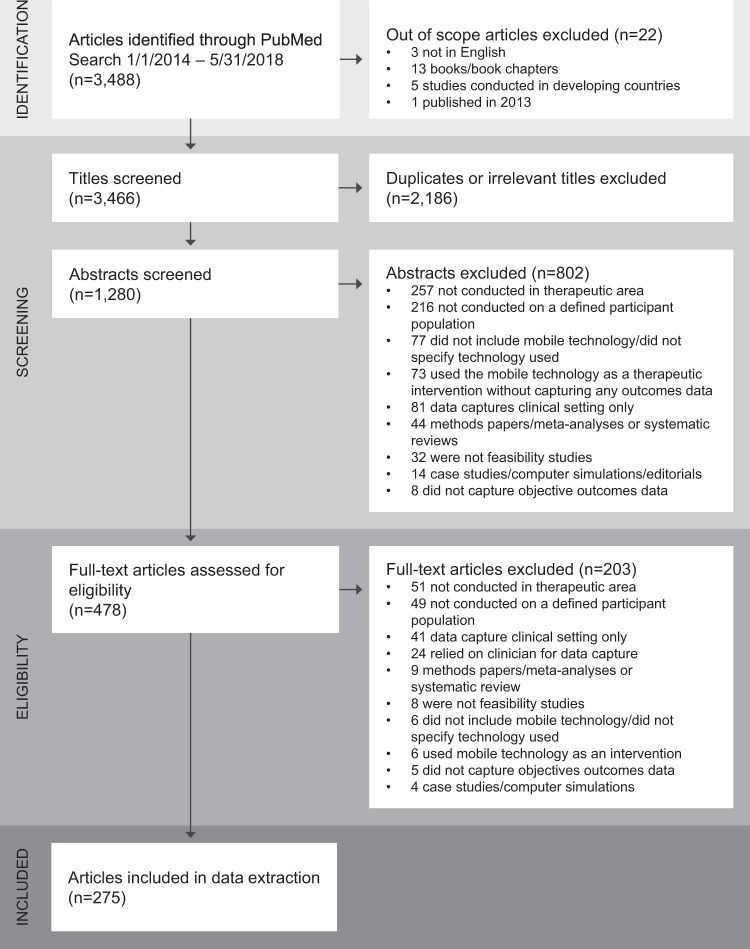
Preferred reporting items for systematic reviews and meta-analyses (PRISMA) flow diagram

Our initial search retrieved 3466 references (see Fig. 1). We excluded over half of the retrieved references (*n* = 2186) after title screening, and abstract screening eliminated a further 63% (*n* = 802). The majority of excluded publications were either not conducted in a defined therapeutic area, or not conducted in a defined participant population. A total of 478 publications were included in the full text review, during which we excluded 203 publications on the basis of our inclusion criteria. Data were extracted from the remaining 275 publications.

### Data categorization

Just over half of all included studies were in neurology or musculoskeletal therapeutic areas, with pulmonary, sleep, endocrine, cardiovascular, and pediatrics making up another 30% combined. Algorithm development was the most common objective (236 studies), followed by sensor performance (133 studies), operational feasibility (126 studies), and software development (24 studies). The median number of participants per study was *n* = 33 (range 1–625), with larger samples evident in studies focused on cardiology, neurology, and musculoskeletal disorders. Two “*n-*of-1’’ studies^11^ were identified (one in nephrology, one in neurology).

Some studies used more than one research tool, such that the 275 studies included 321 technologies. Sixty-seven percent of the technologies were wearable sensors, such as actigraphy, smart-watches, smart-clothing, chest-straps, adhesive patches, and Holter monitors. The remaining tools included tablets and smartphones, implanted sensors such as continuous glucose monitors, and cameras. We did not identify any studies using ingestible sensors. Tablets and smartphones were used in a variety of ways; for example, data captured passively via the embedded accelerometer, and active data capture via an app such as finger-tapping or psychomotor vigilance tasks. Within each of these categories, a wide array of make/model tools were studied, each differing in terms of their sampling frequency, filtering, data processing, and compatible software programs.

In some cases, missing data precluded a full understanding of some studies and this would likely impact reproducibility. Important gaps include the software used for analysis (73% complete), the comparator measure (83% complete), the make and model of the technology (93% complete), and the age and gender of participants (91% and 85%, respectively). All papers reported the number of participants and the type of technology used, although there was substantial variation in the way that sensors were listed in each paper (for example, “motion sensor’’, “accelerometer’’, “tri-axial accelerometer’’). Several papers listed a non-specific term such as “pedometer’’ without specifying the actual sensors contained within. Rather than attempt to impose an interpretation, our database lists the technology as reported within the source publication.

**Fig. 2:**
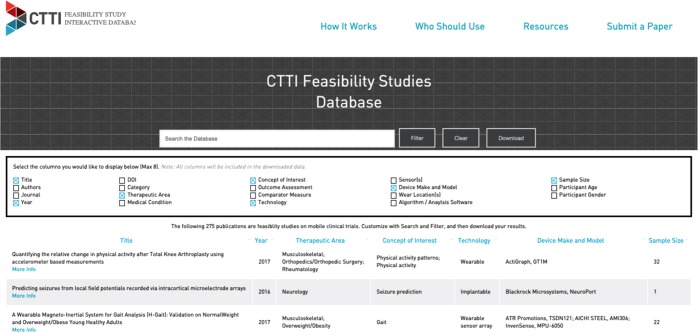
A screenshot of the online database depicting the current layout and features

A static database of all extracted data is accessible via Table e2 (online supplement) with full study references including a digital object identifier (DOI); however, we encourage readers to access the online version that will be updated regularly as more studies emerge (http://feasibility-studies.ctti-clinicaltrials.org). The current layout and features of the online database are shown in Fig. 2. In addition, the papers that we excluded are listed in Table e3. The online version of the database can be filtered and searched electronically, enabling a user to find information most relevant to their work.

## Discussion

In this paper, we describe the methodology underlying our systematic scoping review of feasibility studies focused on mobile technologies. We also summarize key trends that emerged when compiling our searchable database, such as the fact that although some tools we identified (such as Holter monitors and actigraphy) have been used in research and clinical settings for decades, other tools (such as smart-clothing and adhesive patches) are more recent developments, emphasizing that there is still much to learn about different methods of deploying mobile sensors. We noted an absence of standards in both the use of mobile technology in research as well as reporting methodology, evidenced by the lack of consistency across publications, which made data extraction challenging. The development of methodology and reporting standards, although beyond the scope of our current project, would be extremely beneficial for the field. The scope and content of our database demonstrates that the deployment of mobile technology in research is an active, growing area of interest to investigators. Information gleaned from our database can not only be used by sponsors to inform trial design, but may also be useful to regulatory bodies such as the FDA, technology manufacturers, engineers and data scientists, patient groups, institutional review boards and ethics panels, statisticians, health policy planners, and clinicians. Further, the ability to access data from feasibility studies readily is likely to facilitate incorporation of mobile endpoints into settings other than clinical trials, such as observational or interventional health outcomes studies,^12^ translational research,^13^ and eventually, clinical care.^14^

Although specific objectives differed across the publications we identified, in general all studies aimed to determine whether a specific technology and/or outcome assessment was “fit for purpose”; that is, whether the system was capable of generating the necessary data in a stated context of use. Many studies addressed one or both of the following sets of questions: (A) what physical construct is intended to be measured (e.g., movement), what sensor is required to capture those data (e.g., an accelerometer), and how accurate are the sampled data (e.g., intra- and inter-sensor variability of the acceleration signal when compared against a mechanical shaker with known acceleration); *and/or* (B) how are the data converted to a meaningful endpoint (e.g., development of an algorithm that converts an acceleration signal into an estimate of total sleep time) and how does the endpoint perform against a comparator (e.g., the agreement between algorithm-generated sleep data with polysomnography-generated sleep data)? The former set of questions address the concept of verification and relate to intrinsic capabilities of the sensor, whereas the latter address the concept of validation and relate to the application of sensor-derived data to health concepts in human participants. A more thorough explanation of these concepts is included in the CTTI “Advancing the Use of Mobile Technologies for Data Capture and Improved Clinical Trials” recommendations (https://www.ctti-clinicaltrials.org/sites/www.ctti-clinicaltrials.org/files/mobile-devices-recommendations.pdf). Other feasibility studies in our dataset addressed considerations such as ease of use, participant comfort, security and integrity of data transfer or storage, and software development.

We did not attempt to create data fields indicating study quality or exclude papers that did not achieve a certain quality threshold for several reasons. Firstly, there is tension between study quality and reporting quality,^15^ and we were only in a position to evaluate what has been reported. Secondly, we concluded that all of the data we sought to extract were valuable, and that highlighting missing data and variability in reporting would provide an opportunity for investigators in the field to reflect on these issues when preparing future manuscripts. Thirdly, the study quality assessments typically used in other systematic reviews may not be appropriate for feasibility studies. For example, in an algorithm development study aiming to capture steps, the sample size is the number of steps, not the number of participants, although larger participant sample sizes remain important to capture variability and ensure generalizability.^7,8^ In our opinion, it is beneficial to focus on whether a particular feasibility study is useful, rather than whether it is of sufficient quality, although there is likely a wide overlap across these concepts. To that end, we recommend the development of levels of evidence, which may help define what is reported in feasibility studies and will therefore make them more useful to others in the field. Thus, in the absence of methodological and reporting standards, we leave it to end-users to explore the database and compare and contrast what they see with “best practices’’ as outlined in the CTTI Mobile Technology recommendations (https://www.ctti-clinicaltrials.org/projects/mobile-technologies).

One noteworthy area of missing data was the absence of thorough descriptive information regarding study participants. We aimed to capture only what would be considered the most basic demographic information—age and gender/sex—and noted that these variables were missing in 9% and 15% of publications, respectively. Many of the publications in our database were published in journals that typically have an engineering focus; however, all involved data collection in human participants. Although we do not have quantitative data available as we did not aim to extract it, we can attest that many publications captured in our search did not report important participant characteristics such as race/ethnicity, measurements of body habitus, measurements of socioeconomic status, or descriptions of disease severity. The use of mobile technology in clinical studies, particularly those adopting a “bring your own device’’ model, may impose barriers to participation in underrepresented/underserved populations, and therefore we encourage investigators to assess and report the sociodemographic characteristics of study participants, and consider issues of equity and equality during the study design phase.

There are some limitations to our approach that should be noted. Bias may have been introduced by missing relevant literature, given our choice of PubMed as the bibliographic database to search, search terms, and inclusion criteria, particularly given the inconsistencies in terminology across papers. In particular, it is possible that Layer #1 in Table e1, which ensures that a publication refers to some kind of sensor that can be attached to a human participant, may have a slight bias towards technical/engineering authors who may refer to the underlying technology, as opposed to more clinically focused authors who might in some cases use a single term such as a manufacturer name, a device name, or a generic term such as “activity monitor” for wearable technology containing an accelerometer. We attempted to investigate other sources of bias in our search terms; for example, we performed a sensitivity analysis whereby the word “pilot’’ was removed from our search terms, and found that fewer than 5% of eligible publications were missed by doing so. Some methodological decisions may have resulted in the exclusion of papers outside of our scope that some readers might find particularly useful, such as studies conducted entirely in an inpatient or clinical setting, or those published before 2014. Our decision to limit the search to publications from 2014 onwards was because our aim was to assemble a resource that reflects the current state of the art. The contents of the database are limited to those feasibility studies published in the peer-reviewed literature, and we acknowledge that relevant data may also exist in the gray literature, in conference proceedings, or in internal reports used by investigators to inform their own future studies and therefore not published at all. In the future, we hope to develop functionality for the online database so that users can put forward potentially relevant publications that we have missed, as well as unpublished reports.

The use of mobile technologies for data capture is an evolving and rapidly expanding field. CTTI plans to update the literature search annually. This process may require changes to our search terms and data extraction methodology as technology progresses. On a quarterly basis, we will also examine relevant publications that we receive from users of the database that we had missed, to see how our search terms or inclusion criteria might be modified to capture similar publications in the future. Our hope is that the growing interest in this field as well as the demonstrated success of using mobile technology in clinical research, will lead to a more standardized lexicon, as well as relevant medical subject headings (MeSH terms, https://meshb.nlm.nih.gov/) terms that could be assigned to eligible publications, making it easier to find them in future searches. Eventually, if investigators in the field find the online resource useful, journals could encourage or require that authors include all data fields and upload their manuscript to the database, akin to the registration and reporting of clinical trials. Although beyond the scope of the current work, a registry would allow for linking different studies and trials that have adopted the same technology, as well as providing information as to the successful use of mobile technologies in drug approval and/or use in clinical practice.

In conclusion, we have created a freely accessible, online database of feasibility studies assessing the use of mobile technologies for data capture, intended to be a valuable resource for many stakeholder groups, including researchers, ethicists, regulatory bodies, and patient groups. One of our objectives was to create a user-friendly database that investigators in the field can explore as they make decisions regarding which technology would be most useful for a particular research study, although it should be emphasized that clinical relevance is only one part of the decision-making criteria. The CTTI Mobile Technology recommendations provide information on other important topics to consider beyond sensor verification and algorithm validation, such as cyber security, patient preferences, and data rights (https://www.ctti-clinicaltrials.org/programs/mobile-clinical-trials). We hope that the online database resulting from our systematic scoping review reported here becomes a widely used tool, thereby promoting standardization of methodology and reporting, and contributing to the design and implementation of high-quality, efficient trials.

## Methods

### Conduct of the systematic scoping review

On 21 June 2018, CTTI conducted a systematic search of peer-reviewed literature indexed in PubMed and published between January 2014 and May 2018. We did not restrict the scope of our search to any single therapeutic area or mobile technology. A multi-stakeholder team of clinical, academic, technical, operational, and patient experts developed the search terms (listed in Supplementary Table 1 of the online supplement), inclusion criteria (Table 1), and selection of data to be extracted from the final publications (Table 2). A medical librarian supported the development of the search terms.

**Table 1:** Inclusion criteria adopted to enable the identification of suitable feasibility studies

Pre-review	Reported results of original data collection (for example, meta-analyses, editorials, letters, opinion pieces, and methods papers were excluded).
Population	Collected data from human participants (for example, studies that reported results of a computer simulation were excluded).
	Stated a specific therapeutic area.
	Defined a participant population that either:
	a. Included participants from the target population *or;* b. Included participants that would be generalizable to the target population.
Intervention	Included at least one mobile technology meeting our definition for objective outcome (efficacy or safety) data capture.
	Defined the specific technology used.
Comparator	Specified a comparator (sensor performance and algorithm development studies only).
Outcome	Evaluated mobile technology/ies capturing objective outcomes data (for example, studies examining as the primary technology were excluded).
	When mobile technology/ies were used as a therapeutic intervention, the study reported outcomes data.
Study design	Described a feasibility study in line with our definition; specifically, a feasibility study addresses one or more of the following components: a. Performance of an outcome of interest against a comparator where the outcome of interest could be related to: i. Measurement performance of sensor *and/or;* ii. Algorithm performance (clinical endpoints); b. Human factors considerations (acceptability, tolerability and usability); c. Participant adherence; d. Completeness of data.
	Captured data outside of a clinical setting *or* captured data in an inpatient or clinic setting specifically to enable out-of-clinic use.
	Reported data from a participant sample (for example, case studies were excluded; however, n-of-1 studies^12^ were considered in scope).
	Country of origin is reported to have “high’’ or “very high’’ human development by the United Nations Human Development Index, http://hdr.undp.org/en/composite/HDI.

*ePRO* electronic patient-reported outcome

**Table 2:** Data fields extracted from identified feasibility studies

Field	Definition	Allowed values
Title		Free text
Authors	Last name, initials	Free text
Journal	Name	Free text
Year		2014, 2015, 2016, 2017, 2018
DOI	Digital object identifier. A unique alphanumeric string used to identify content and provide a persistent link to the manuscript’s online location.	Free text
Category	The type of study according to the authors’ objectives.	Sensor performance, algorithm development, operational feasibility, software development
Therapeutic area	A knowledge field that focuses on research and development of treatments for diseases and pathologic findings, as well as prevention of conditions that negatively impact the health of an individual.	Selected from a list of FDA-approved drugs by therapeutic area, https://www.centerwatch.com/drug-information/fda-approved-drugs/therapeutic-areas, with “pre-natal’’ included as an additional therapeutic area.
Medical condition	An abnormal state of health that interferes with normal or regular feelings of wellbeing.	Free text
Concept of interest	The aspect of an individual’s clinical, biological, physical, or functional state, or experience that the assessment is intended to capture (or reflect).	Free text
Outcome assessment	The measureable characteristic that is influenced or affected by an individuals’ baseline state or an intervention as in a clinical trial or other exposure.	Free text
Comparator measure	The measure used to benchmark the digital measure against.	Free text
Technology	A description of the sensor casing and modality as experienced by the participant.	Adhesive patch, camera, chest strap, continuous glucose monitor, holter monitor, implantable, smart clothing, smart phone, smart shoe, smart watch, tablet, wearable, Wearable sensor array.
Sensor(s)	The component of the technology that detects or measures a physical property and records, indicates, or otherwise responds to it.	Free text
Make, model manufacturer	The make, model and manufacturer of the technology.	Free text
Wear location	Where the technology is positioned on the participant’s body.	Free text
Algorithm/ analysis software	Name and version.	Free text
Sample size	Total number of participants in the feasibility study.	*N*
Participant age	Infants <1year	Infants, children, adolescents, adults, older adults
	Children 1–10	
	Adolescent 11–17	
	Adult 18–64	
	Older adult 65+	
Participant gender	Gender or sex.	Male, female, both, unknown

Following the PubMed search, we conducted a multi-step review process to select publications for inclusion. First, two of four trained analysts (A.B., E.B., C.M., K.W.) independently reviewed each publication title against the inclusion criteria, following the PICOS^16^ (Population, intervention, comparison, outcome; study design) framework. Second, two of the four analysts reviewed the abstracts of the remaining, potentially eligible publications to determine whether each met our inclusion criteria. When there was disagreement between two reviewers during either phase, the decision whether to advance a publication was resolved by a third analyst. Finally, two analysts reviewed the full text of each of the publications that passed the abstract screening stage, with a third used to settle any disagreements and establish the final list of publications for inclusion.

To build the database, four analysts (A.B., E.B., C.M., K.W.) extracted the data and categorized each publication as described in Table 2. Each publication was assigned to one or more of the following categories: sensor performance; algorithm development; operational feasibility; and software development. The following data were extracted from each: medical condition (used to identify therapeutic area); concepts of interest captured by the mobile technology (for example, sleep); specific outcomes captured by the digital measurement (for example, total sleep time); comparator used to assess the digital measurement (for example, polysomnography); information relating to the sensor/s (for example, accelerometry and photoplethysmography); details related to the algorithms or software (if applicable); and descriptive data for the study sample. After the data were extracted from each publication, it was standardized by two analysts (J.G. and C.M.).

### Reporting Summary

Further information on research design is available in the Nature Research Reporting Summary linked to this article.

## Supplementary information

SI

Reporting Summary

Supplementary Data 1

Supplementary Data 2

## Data Availability

All data generated or analyzed during this study are included in this published article (and its supplementary information files).

## Supplementary information

**Supplementary information** accompanies the paper on the *npj Digital Medicine* website (10.1038/s41746-019-0125-x).

## References

[1] Perry B (2018). Use of mobile devices to measure outcomes in clinical research, 2010–2016: a systematic literature review. Digit. Biomark..

[2] Arnerić SP (2017). Biometric monitoring devices for assessing end points in clinical trials: developing an ecosystem. Nat. Rev. Drug Discov..

[3] Wright SP, Brown TSH, Collier SR, Sandberg K (2017). How consumer physical activity monitors could transform human physiology research. Am. J. Physiol.-Regul., Integr. Comp. Physiol..

[4] Wright SP, Collier SR, Brown TS, Sandberg K (2017). An analysis of how consumer physical activity monitors are used in biomedical research. FASEB J..

[5] Banaee H, Ahmed MU, Loutfi A (2013). Data mining for wearable sensors in health monitoring systems: a review of recent trends and challenges. Sens. (Basel, Switz.).

[6] Cohen AB, Mathews SC (2018). The digital outcome measure. Digit. Biomark..

[7] Izmailova ES, Wagner JA, Perakslis ED (2018). Wearable devices in clinical trials: hype and hypothesis. Clin. Pharmacol. Ther..

[8] Dodge HH (2015). Use of high-frequency in-home monitoring data may reduce sample sizes needed in clinical trials. PLoS ONE.

[9] Treweek S (2015). Making randomised trials more efficient: report of the first meeting to discuss the Trial Forge platform. Trials.

[10] Ioannidis JP (2014). Increasing value and reducing waste in research design, conduct, and analysis. Lancet (Lond., Engl.).

[11] Lillie EO (2011). The n-of-1 clinical trial: the ultimate strategy for individualizing medicine?. Pers. Med..

[12] Speier W (2018). Evaluating utility and compliance in a patient-based eHealth study using continuous-time heart rate and activity trackers. J. Am. Med. Inform. Assoc..

[13] Dhawan AP (2018). Editorial trends and challenges in translation of point-of-care technologies in healthcare. IEEE J. Transl. Eng. Health Med..

[14] Majumder S, Mondal T, Deen MJ (2017). Wearable sensors for remote health monitoring. Sensors (Basel).

[15] Soares HP (2004). Bad reporting does not mean bad methods for randomised trials: observational study of randomised controlled trials performed by the Radiation Therapy Oncology Group. BMJ (Clin. Res.).

[16] Schardt C, Adams MB, Owens T, Keitz S, Fontelo P (2007). Utilization of the PICO framework to improve searching PubMed for clinical questions. BMC Med. Inform. Decis. Mak..

