# Supplementary material for: A systematic review of feasibility studies promoting the use of mobile technologies in clinical research
Source: NPJ Digit Med. 2019 Jun 6;2:47. doi: 10.1038/s41746-019-0125-x (PMC6554345; doi:10.1038/s41746-019-0125-x)
Supplement: Supplementary file 1 — SI [file 41746_2019_125_MOESM1_ESM.docx]

**Supplementary Table 1: PubMed search terms used in the systematic review**

| **Layer** | **Search Terms** | **Results Returned (search date June 21, 2018)** |
| --- | --- | --- |
| #1 | ("instrumentation"[Subheading] OR "Transducers"[Mesh] OR "Signal Processing, Computer-Assisted"[Mesh] OR accelerometer[tiab] OR accelerometers[tiab] OR gyroscope[tiab] OR gyroscopes[tiab] OR instrumentation[tiab] OR instrument[tiab] OR instruments[tiab] OR instrumented[tiab] OR interface[tiab] OR device[tiab] OR devices[tiab] OR equipment[tiab] OR transducer[tiab] OR transducers[tiab] OR biochip[tiab] OR biochips[tiab] OR sensor[tiab] OR sensors[tiab] OR sensoring[tiab] OR sensorized[tiab] OR biosensor[tiab] OR biosensors[tiab]) AND (wearable[tiab] OR wearing[tiab] OR wear[tiab] OR wears[tiab] OR wore[tiab] OR worn[tiab] OR contact[tiab] OR contacts[tiab] OR "non-contact"[tiab] OR skin[tiab] OR adhere[tiab] OR adheres[tiab] OR implant[tiab] OR implants[tiab] OR implanted[tiab] OR ingestible[tiab] OR ingests[tiab] OR ingested[tiab] OR portable[tiab] OR embedded[tiab]) | 149,838 |
| #2 | (test[tiab] OR tests[tiab] OR testing[tiab] OR tested[tiab] OR measure[tiab] OR measures[tiab] OR measured[tiab] OR measuring[tiab] OR detect[tiab] OR detects[tiab] OR detection[tiab] OR monitor[tiab] OR monitors[tiab] OR monitoring[tiab] OR recorded[tiab] OR records[tiab] OR recording[tiab]) AND (compare[tiab] OR compares[tiab] OR compared[tiab] OR comparison[tiab] OR validate[tiab] OR validates[tiab] OR validated[tiab] OR reproduce[tiab] OR reproduces[tiab] OR reproducibility[tiab] OR equivalent[tiab] OR assessment[tiab] OR assesses[tiab] OR assessed[tiab] OR predict[tiab] OR predicts[tiab] OR predicted[tiab] OR prediction[tiab]) AND ("Monitoring, Physiologic"[Mesh] OR "Accelerometry"[Mesh] OR "Physical Examination"[Mesh] OR "Movement"[Mesh] OR "physiology"[subheading] OR "physiopathology"[subheading] OR "Signs and Symptoms"[Mesh] OR accelerometry[tiab] OR actigraphy[tiab] OR movement[tiab] OR gait[tiab] OR pathology[tiab] OR pathologies[tiab] OR physiopathology[tiab] OR physiopathologies[tiab] OR physiological[tiab] OR physiologic[tiab] OR function[tiab] OR functional[tiab] OR activity[tiab] OR activities[tiab] OR locomotion[tiab] OR consumption[tiab] OR exercise[tiab] OR exercises[tiab] OR health[tiab]) | 1,789,427 |
| #3 | "Algorithms"[Mesh] OR algorithm[tiab] OR algorithms[tiab] OR "Reproducibility of Results"[Mesh] OR reproducible[tiab] OR reproducibility[tiab] OR reliability[tiab] OR reliable[tiab] OR validity[tiab] OR validation[tiab] OR validates[tiab] OR accuracy[tiab] OR accuracies[tiab] OR feasibility[tiab] OR precision[tiab] OR precise[tiab] OR "gold standard"[tiab] OR "gold standards"[tiab] OR "Sensitivity and Specificity"[Mesh] OR sensitivity[tiab] OR specificity[tiab] OR "signal-to-noise"[tiab] OR "detection limit"[tiab] OR "detection limits"[tiab] OR "limits of detection"[tiab] OR (("clinical trial"[tiab] OR "clinical trials"[tiab]) AND ("use in"[tiab] OR "used in"[tiab])) | 2,754,520 |
| #4 | #1 AND #2 AND #3 | 9,629 |
| #5 | #4 NOT (Editorial[ptyp] OR Letter[ptyp] OR Case Reports[ptyp] OR comment[ptyp]) NOT (animals[mh] NOT humans[mh]) | 8,673 |
| #6 | #5 AND (("2014/01/01"[Date - Publication]: "3000"[Date - Publication])) | 3,488 |

PubMed indexing abbreviations: MeSH= Medical Subject Headings®, MH = MeSH Heading; ptyp = publication type; tiab = restricts query to search in the title or abstract of the articles.
